# Methamphetamine use increases the risk of cerebral small vessel disease in young patients with acute ischemic stroke

**DOI:** 10.1038/s41598-023-35788-z

**Published:** 2023-05-25

**Authors:** Zhu Zhu, Benjamin Vanderschelden, Sook Joung Lee, Haley Blackwill, Mohammad Shafie, Jennifer E. Soun, Daniel Chow, Peter Chang, Dana Stradling, Tianchen Qian, Wengui Yu

**Affiliations:** 1 Department of Neurology, University of California Irvine, 200 S. Manchester Ave. Suite 206, Orange, CA 92868 USA; 2 Department of Radiological Science, University of California, Irvine, CA USA; 3 Department of Statistics, University of California, Irvine, CA USA; 4 Department of Physical Medicine and Rehabilitation, College of Medicine, The Catholic University of Korea, Seoul, Republic of Korea

**Keywords:** Neuroscience, Neurology

## Abstract

Methamphetamine use causes spikes in blood pressure. Chronic hypertension is a major risk factor for cerebral small vessel disease (cSVD). The aim of this study is to investigate whether methamphetamine use increases the risk of cSVD. Consecutive patients with acute ischemic stroke at our medical center were screened for methamphetamine use and evidence of cSVD on MRI of the brain. Methamphetamine use was identified by self-reported history and/or positive urine drug screen. Propensity score matching was used to select non-methamphetamine controls. Sensitivity analysis was performed to assess the effect of methamphetamine use on cSVD. Among 1369 eligible patients, 61 (4.5%) were identified to have a history of methamphetamine use and/or positive urine drug screen. Compared with the non-methamphetamine group (n = 1306), the patients with methamphetamine abuse were significantly younger (54.5 ± 9.7 vs. 70.5 ± 12.4, *p* < 0.001), male (78.7% vs. 54.0%, *p* < 0.001) and White (78.7% vs. 50.4%, *p* < 0.001). Sensitivity analysis showed that methamphetamine use was associated with increased white matter hyperintensities, lacunes, and total burden of cSVD. The association was independent of age, sex, concomitant cocaine use, hyperlipidemia, acute hypertension, and stroke severity. Our findings suggest that methamphetamine use increases the risk of cSVD in young patients with acute ischemic stroke.

## Introduction

Methamphetamine abuse has emerged as a risk factor for both hemorrhagic and ischemic stroke in recent years^1–10^. Although population-based study and forensic analysis of fatal strokes showed significant predominance of hemorrhagic stroke in methamphetamine users^3,6,7^, methamphetamine abuse has also been increasingly reported to be associated with acute ischemic stroke (AIS)^1,2,4–6^.

The mechanisms by which methamphetamine causes stroke are still unknown. Case studies and forensic analysis showed atherosclerotic stenoses, arterial dissection, and berry aneurysms in patients with methamphetamine-associated stroke^2,4,6,7^. Methamphetamine abuse was also shown to produce a dose-dependent elevation of blood pressure and chronic hypertension^11–13^.

Chronic hypertension is a major risk factor for cerebral small vessel disease (cSVD) and stroke^13–16^. cSVD refers to a group of pathological processes that affect the small perforating vessels and capillaries in the brain^16,17^. Radiographically, it is characterized by deep white matter hyperdensities (WMHs), lacunar infarcts, microbleeds, and enlarged perivascular spaces (PVS) on magnetic resonance imaging (MRI) of the brain^17^. cSVD is a common cause of stroke, cognitive impairment, and vascular dementia^15–17^.

The aim of this study is to investigate whether methamphetamine abuse increases the risk of cSVD in patients with acute ischemic stroke.

## Methods

This retrospective study was approved by the University of California Irvine Institutional Review Board (IRB) and the Ethics Committee. Informed consents were waived due to retrospective study design and minimal harm to the patients. All methods in the study were performed in accordance with the relevant guidelines and regulations.

### Study population

Consecutive AIS patients admitted at the University of California Irvine Medical Center from January 1, 2013 to December 30, 2018 were included. The patient list was generated from the prospectively maintained American Heart Association (AHA)'s *Get-With-The-Guideline* stroke data registry at our medical center. The registry uses a web-based patient management tool to collect clinical data on consecutively admitted patients, to provide decision support, and to enable real-time online reporting^18^. Patients with TIA, stroke mimics, subacute stroke, inpatient stroke, and primary brain hemorrhage were excluded. Stroke transfers from outside facilities were also excluded.

All patients underwent standard diagnostic evaluation and treatment per AHA guidelines^19^. Based on the history of methamphetamine abuse and/or urine drug screen (UDS), patients were divided into Meth and Non-Meth group. The UDS was performed using EMIT II Plus Amphetamines Assay (Beckman Coulter, Inc) with a sensitivity and specificity of 94.3% and 93.3%, respectively^20^.

### Study parameters

MRI images of the brain were reviewed by an experienced neurologist (Zhu Z) to assess cSVD using modified Fazekas scale^13–15,21^. Both deep and periventricular WMHs were rated from 0 to 3 on fluid-attenuated inversion recovery (FLAIR)- and T2-weighted sequences^15^. Lacunes were defined as small (< 15 mm) subcortical infarcts^13,21^. Cerebral microbleeds (CMBs) were rated on susceptibility-weighted imaging (SWI) based on their numbers (< 5, 5–10, or > 10). Enlarged perivascular spaces (PVS) were defined as small (< 3 mm) punctate hyperintensities on FLAIR or T2-weighted images and rated from 0 to 3: 0 (absent), 1 (< 10), 2 (10–25), and 3 (> 25).

Total burdens of cSVD were rated from 0 to 4^13^. One point was added for each of the following findings: confluent deep or periventricular WMHs (Fazekas grade 2 and 3), ≥ 1 lacune, ≥ 1 CMBs, and > 10 enlarged PVS in the basal ganglia on at least one side of the brain.

### Propensity score matching

Propensity score matching with a 1:1 ratio was performed to select patients from the Non-Meth group as control. The propensity score was estimated using a logistic regression model based on age, sex, hypertension, diabetes mellitus, hyperlipidemia, obesity, and initial NIHSS score as described (1:1 match, nearest neighbor approach)^22^.

### Statistical analysis

Continuous variables were described by mean ± standard deviation (SD) or median with interquartile range (IQR) based on the results of normality testing. Categorical variables were expressed by counts with percentages. Baseline characteristics and outcome at discharge were compared between Meth and Non-Meth groups by Mann Whitney test for continuous variables and chi-square test for categorical variables. Sensitivity analysis was performed to investigate the effect of methamphetamine abuse and other variables on the development of cSVD. All statistical analyses were performed using SPSS software (version 23.0). A 2-tailed value of *p* < 0.05 was considered statistically significant. All statistical analyses were reviewed and verified by a biostatistician (Qian T).

## Results

**Figure 1:**
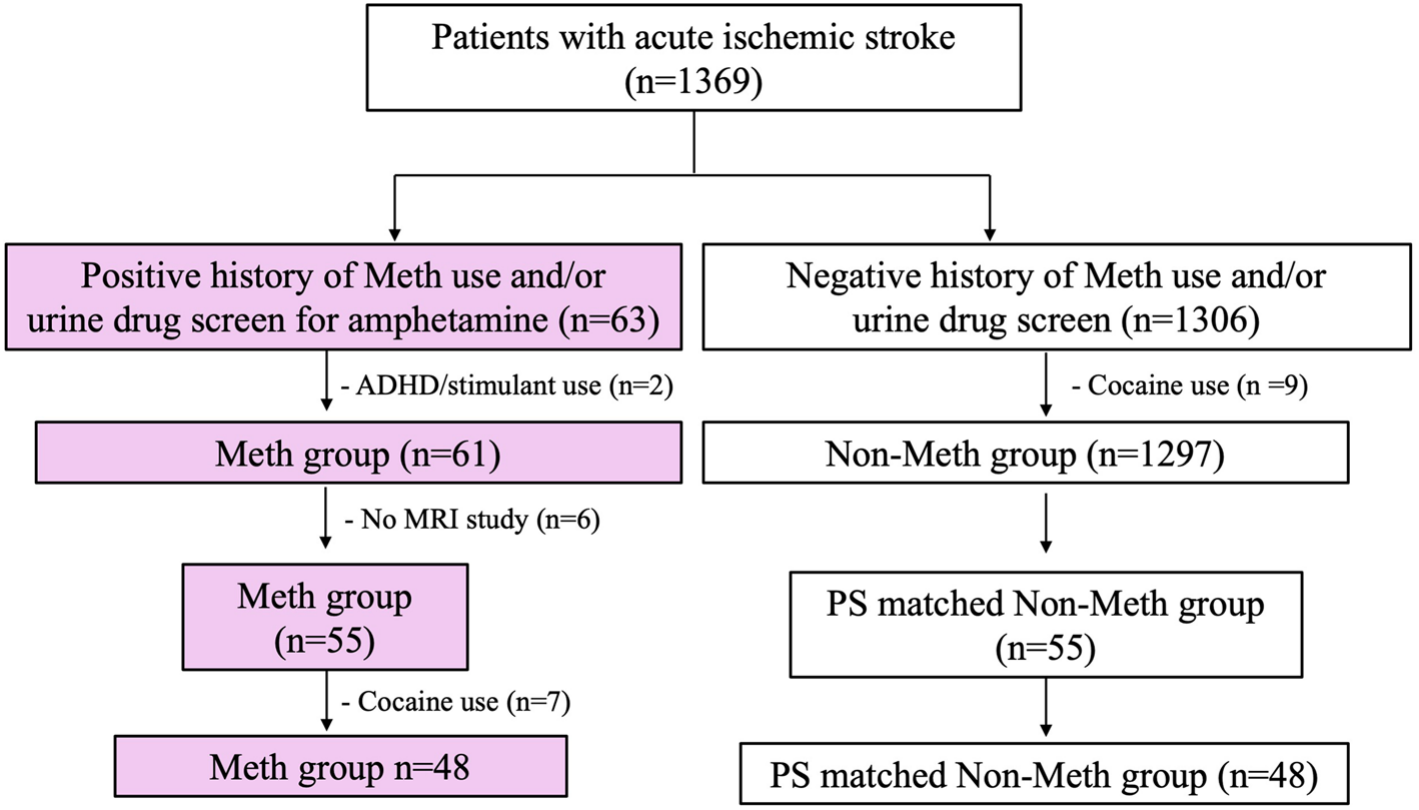
Study flowchart.

From the 1,369 patients with AIS, 63 were found to have a history of methamphetamine abuse or positive UDS. 2 patients were identified to have a positive UDS due to stimulant use for Attention Deficit Hyperactivity Disorders (ADHD) and were excluded from the study. Of the 61 patients in the Meth group, 6 had no MRI study of the brain and were excluded for the investigation of cSVD. In the remaining 55 patients, 7 had concomitant cocaine use and were excluded during the final statistical analysis. In patients with a negative history of meth use or UDS, 9 had cocaine use and was excluded. A 1:1 propensity score-matched control group was selected from the Non-Meth group for sensitivity analysis. The study flowchart is shown in Fig. 1.

The characteristics of the Meth and Non-Meth groups are summarized in Table 1. Compared with Non-Meth group, patients in the Meth group were significantly younger (54.5 ± 9.7 vs. 70.5 ± 12.4, *p* < 0.001), more likely male (78.7% vs. 54.0%, *p* < 0.001) and White (78.7% vs. 50.4%, *p* < 0.001). There appeared to be proportionally less Asians with methamphetamine-associated stroke (3.3% vs. 24.1%, *p* < 0.001).

**Table 1:** Demographics and clinical characteristics in the Meth group versus Non-meth group.

Variables	Meth group	Non-meth group	*p*
n	61	1297	–
Age	54.5 ± 9.7	70.5 ± 12.4	** < 0.001**
Male (%)	48 (78.7%)	709 (54.0%)	** < 0.001**
Race			
White	48 (78.7%)	654 (50.4%)	** < 0.001**
Hispanic	6 (9.8%)	231 (17.8%)	0.197
Black	3 (4.9%)	30 (2.3%)	0.213
Asian	2 (3.3%)	312 (24.1%)	** < 0.001**
Other	2 (3.3%)	70 (5.4%)	0.471
Hypertension	35 (57.4%)	935 (71.4%)	**0.013**
Diabetes	20 (32.8%)	447 (36.4%)	0.788
Hyperlipidemia	16 (26.2%)	523 (40.0%)	**0.028**
Obesity	10 (16.4%)	178 (13.6%)	0.555
Smoking	35 (57%)	390 (30.1%)	** < 0.001**
Cocaine use	7 (11.5%)	9 (0.7%)	** < 0.001**
Statin use	10 (16%)	525 (40.5%)	** < 0.001**
Antithrombotic use	14 (23%)	452 (34.8%)	0.056
Initial NIHSS score	4 (2, 10)	4 (2, 14)	0.154
Baseline LDL cholesterol	104 ± 38	99 ± 39	0.213
Mechanism of stroke			
Large artery disease	15 (25%)	312 (24.1%)	0.924
Cardioembolism	21 (34%)	592 (45.6%)	0.085
Small vessel disease	19 (31%)	361 (27.8%)	0.573
Other causes	2 (3%)	52 (4%)	0.775

*NIHSS* National Institutes of Health Stroke Scale, *LDL* low density lipoprotein cholesterol.

Significant are in value [bold].

Non-Meth group had a statistically significant higher rate of hypertension, hyperlipidemia, and statin use than in the Meth group. There was no statistically significant difference between the two groups in the history of diabetes, obesity, antithrombotic use, initial NIHSS score, baseline LDL cholesterol levels, or mechanisms of stroke per TOAST classification (Table 1). However, the patients in the Meth group had a higher rate of smoking (57% vs. 30.1%, *p* < 0.001) or cocaine use (11.5% vs. 0.7%, p < 0.001) than those in the Non-Meth group.

### Sensitivity analysis

Sensitivity analysis was performed to investigate the effect of methamphetamine use as independent variable. Compared with age- and sex-matched control group, there were proportionally more White and less Asian patients in the Meth group (Table 2). There was no significant difference in the history of hypertension, diabetes, hyperlipidemia, obesity, or initial NIHSS score between the Meth group and propensity score matched control group.

**Table 2:** Comparison of cerebral SVD in Meth versus propensity score-matched control group after removing the patients with stimulant use for ADHD.

Variables	Meth group	PS-matched control group	*p*
N	55	55	
Age	55 ± 10	55 ± 10	0.978
Male (%)	45 (81.8)	38 (69.1)	0.183
Race			**0.006**
White	43 (78.2)	25 (45.5)	
Hispanic	6 (10.9)	4 (7.3)	
Black	3 (5.5)	11 (20)	
Asian	2 (3.6)	10 (18.2)	
Other	1 (1.8)	5 (9.1)	
Hypertension	36 (65.5)	31 (56.4)	0.329
Diabetes	19 (34.5)	20 (36.4)	0.329
Hyperlipidemia	15 (27.3)	20 (36.4)	0.306
Obesity	10 (18.2)	13 (23.6)	0.482
NIHSS	5 (6)	4 (6)	0.245
Cerebral SVD			
WMHs	3 (3)	2 (3)	** < 0.001**
Lacunes	2 (4)	1 (2)	**0.032**
Microbleeds	2 (4)	0 (4)	0.369
Enlarged perivascular space	1 (1)	1 (1)	0.346
Total burden	2 (1)	2 (1)	**0.006**
Baseline SBP	159 ± 31	170 ± 39	0.083
Baseline DBP	97 ± 20	95 ± 20	0.573
SBP at discharge	130 ± 17	133 ± 20	0.411
DBP at discharge	77 ± 13	76 ± 13	0.703
Reduction in SBP	27 ± 27	40 ± 30	**0.038**
Reduction in DBP	19 ± 21	20 ± 20	0.915
Numbers of Antihypertensives	3 (3)	2 (3)	0.790

The Meth group in this analysis excluded patients with history of stimulant use for ADHD. Propensity score-matched patients from the Non-Meth group was used as control.

Data are expressed as n (%), mean ± SD, or median (interquartile range, IQR).

*ADHD* attention deficit hyperactivity disorder, *DBP* diastolic blood pressure, *NIHSS* National Institutes of Health Stroke Scale, *PS* propensity score, *SBP* systolic blood pressure, *WMHs* white matter hyperdensities.

Significant are in value [bold].

**Figure 2:**
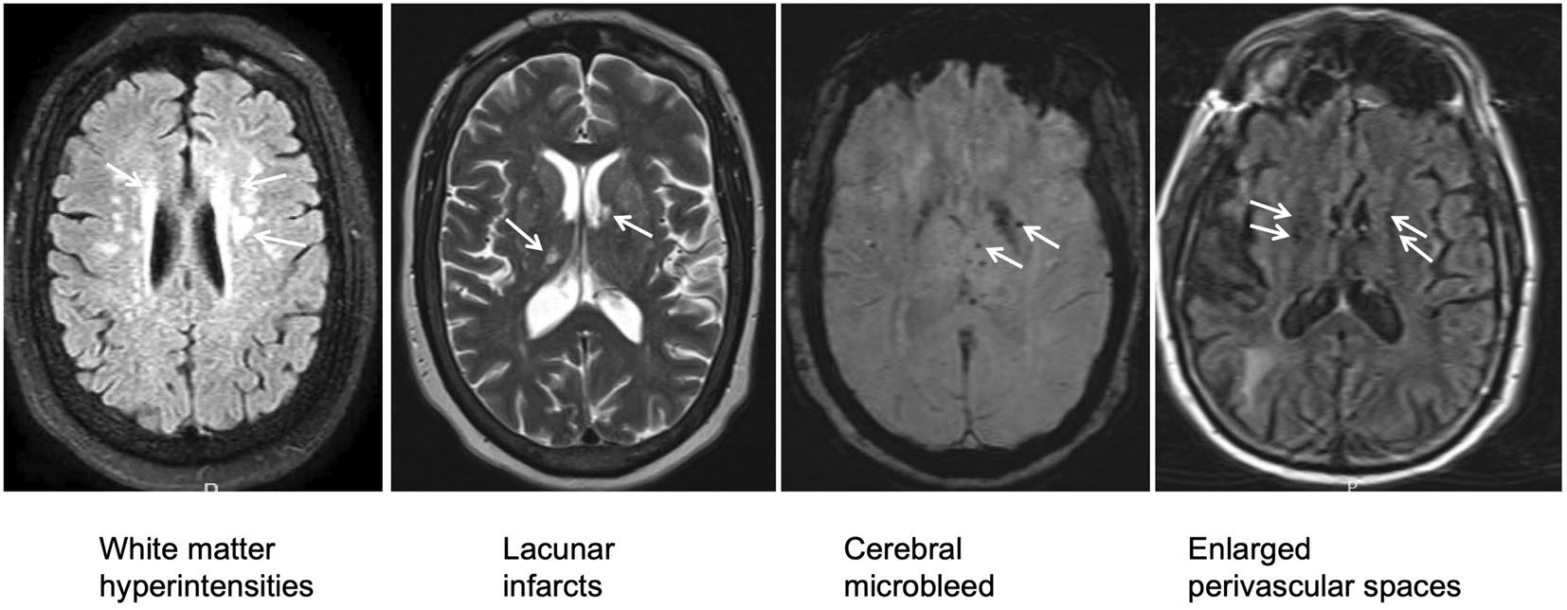
Representative images of cerebral small vessel disease (cSVD). One point is added for each of the following findings to estimate cSVD score: severe white matter hyperintensities (periventricular Fazekas grade 2 or 3); ≥ 1 lacunar infarcts; ≥ 1 cerebral microbleed; and > 10 perivascular spaces visible in the basal ganglia on at least one side of the brain.

The representative MRI images of cSVD were shown in Fig. 2. The Meth group were found to have more WMHs, lacunes, and total burdens of cSVD than propensity score-matched control group (Table 2).

There were no significant differences in systolic or diastolic blood pressure (BP) at admission or hospital discharge, DBP reduction during hospitalization, and numbers of antihypertensives required for BP control between the Meth and propensity score-matched control group. The statistically more significant reduction in SBP in the control group was likely due to higher baseline SBP than in the Meth group (Table 2). These results suggested that the increased burdens of cSVD in the Meth group are independent of age, sex, comorbidities, acute hypertension, and stroke severity as measured by initial NIHSS scores.

We performed an additional sensitivity analysis after excluding the 7 patients with concomitant cocaine use in the Meth groups. As showed in Table 3, the results were consistent with what were shown in Table 2. Methamphetamine use remains an independent predictor for cSVD.

**Table 3:** Comparison of cerebral SVD in the Meth group versus PS-matched control group after removing the patients with stimulant use for ADHD and cocaine use.

Variables	Meth	PS-matched Control	*p*
N	48	48	
Age	54 ± 10	54 ± 10	0.984
Male (%)	34 (70.8)	39 (81.3)	0.232
Race			**0.019**
White	37 (77.1)	22 (45.8)	
Hispanic	3 (6.3)	3 (6.3)	
Black	5 (10.4)	10 (20.8)	
Asian	2 (4.2)	8 (16.7)	
Other	1 (2.1)	5 (10.4)	
Hypertension	31 (64.6)	25 (52.1)	0.214
Diabetes	18 (37.5)	20 (41.7)	0.676
Hyperlipidemia	14 (29.2)	17 (35.4)	0.513
Obesity	9 (18.8)	11 (22.9)	0.615
NIHSS	5 (6)	4 (7)	0.116
Cerebral SVD			
WMHs	3 (3)	2 (3)	** < 0.001**
Lacunes	2 (4)	1 (2)	**0.016**
Microbleeds	1 (4)	0 (3)	0.445
Enlarged perivascular space	1 (1)	1 (1)	0.206
Total burden	2 (2)	2 (1)	**0.009**
Baseline SBP	159 ± 31	172 ± 35	0.070
Baseline DBP	90 ± 20	96 ± 20	0.691
SBP at discharge	132 ± 17	133 ± 20	0.838
DBP at discharge	78 ± 13	76 ± 13	0.481
Reduction in SBP	27 ± 25	40 ± 30	**0.029**
Reduction in DBP	19 ± 20	20 ± 19	0.756
Numbers of Antihypertensives	1 (2)	2 (3)	0.985

The Meth group in this analysis excluded the patients with history of ADHD/stimulant use or cocaine use. Propensity score-matched patients from the Non-Meth group was used as control.

Data are expressed as n (%), mean ± SD, or median (interquartile range, IQR).

*DBP* diastolic blood pressure, *NIHSS* National Institutes of Health Stroke Scale, *PS* propensity score, *SBP* systolic blood pressure, *WMHs* white matter hyperdensities.

Significant are in value [bold].

## Discussion

This single center data demonstrates that methamphetamine abuse is seen in 4.5% of the patients with acute ischemic stroke at our medical center. The patients with methamphetamine use are significantly younger (54.5 ± 9.7 vs. 70.5 ± 12.4, *p* < 0.001) and more likely male (78.7% vs. 54.0%, *p* < 0.001). In addition, sensitivity analysis with propensity score-matched controls showed that methamphetamine abuse is associated with increased burdens of cSVD, independent of age, sex, cocaine use, comorbidities, acute hypertension, and stroke severity.

Previous studies showed that elevated blood pressure levels are associated with each of the MRI markers of cSVD^14,15^. Effective treatment of hypertension may reduce the rates of cSVD and stroke^13,15^. In this retrospective study, there was no significant difference in blood pressures at admission or hospital discharge, blood pressure reduction during hospitalization, and the numbers of antihypertensives required to control hypertension between the Meth or Meth + group and propensity score-matched Non-Meth controls. It is possible that chronic hypertension from methamphetamine abuse plays an important role in the development of cSVD^13^.

Advanced age and male sex were reported to be major risk factors for cSVD^16,23–25^. In a recent study, cerebral SVD was seen in 18.9% of age group 70s as compared to 3% in age group 40s^23^. In a Chinese population-based study, advanced age was shown to be independently associated with the prevalence of cSVD^24^. In our study, we demonstrated that methamphetamine abuse increases the risk of cSVD in young stroke patients.

In a cohort study of homeless and unstably housed women (n = 30) from San Francisco community, 86% patients had a history of cocaine use and 54% patients had WMHs^25^. In our cohort, more patients in the Meth group had a history of cocaine abuse than the Non-Meth group (11.5% vs. 0.7%). After excluding patients with cocaine use in both Meth and Non-Meth groups, methamphetamine use remains an independent predictor for cSVD.

Although the Non-Meth group had statistically significant higher rate of hyperlipidemia (40.0% vs. 26.2%, *p* = 0.028) and statin use (40.5% vs. 15%, *p* < 0.001) than the Meth group, there was no statistically significant difference in baseline LDL cholesterol levels between the 2 groups. In the sensitivity analysis with propensity-score matched control, Meth use remains an independent predictor of cSVD after adjusting for hyperlipidemia.

There were also significantly more patients with a history of smoking in the Meth group than in the Non-Meth group (57% vs. 30%, *p* < 0.001). Since previous study demonstrated a dose-dependent association between pack-years of smoking and WMH progression^26^, smoking is likely a confounding factor in our retrospective study.

There was no statistically significant difference in stroke mechanisms between the Meth and Non-meth group. The Non-meth group had a trend of more cardioembolic stroke, likely due to significantly older ages. There was only one case of possible Reversible Cerebral Vasoconstriction Syndrome (RCVS) as the possible stroke mechanism in the Meth group.

Since cSVD increases the risk of cognitive impairment and vascular dementia, methamphetamine abuse in young adults may have more significant long-term public health concerns than stroke and other detrimental effects.

Our study has a few limitations. First, our data suggested a possible association and cannot prove causal relationship between methamphetamine use and cSVD. Additional studies are required to corroborate our findings. Second, some patients were unable to provide history of drug use due to aphasia or severe neurological deficit. Only 932 patients (71%) in the Non-Meth group had a UDS. The rate of methamphetamine abuse was likely underestimated. Third, there was no information regarding the route, frequency, and duration of methamphetamine abuse. Lastly, smoking, and other confounding factors cannot be ruled out in this retrospective study. Further studies are warranted to adjust for all confounding factors and to investigate the temporal relationship between methamphetamine abuse and cSVD.

Of note, it was challenging to get accurate information on polysubstance abuse, particularly, in patients with neurological deficit. It may be also unethical to conduct randomized controlled studies on methamphetamine abuse. Well-designed prospective registry may be a good option to further investigate the effect of chronic methamphetamine use on cSVD.

In conclusion, our preliminary results demonstrates that methamphetamine abuse is common in young adults with acute ischemic stroke and increases the risk of cSVD. Given increased prevalence of methamphetamine abuse in young adults, additional studies are warranted to investigate the effects of chronic methamphetamine use on the pathogenesis of stroke, cSVD, and vascular dementia.

## Data Availability

Data of this study are available from the corresponding author on reasonable request.
